# Algorithmic management: psychological measurement and associations with work design and mental strain

**DOI:** 10.1186/s40359-025-03680-2

**Published:** 2025-12-01

**Authors:** Carsten Röttgen, Hendrik Dzaack, Britta Herbig, Tobias Weinmann, Andreas Müller

**Affiliations:** 1 Institute of Psychology, Work and Organizational Psychology, University of Duisburg-Essen, Essen, Germany; 2 Institute and Clinic for Occupational, Social and Environmental Medicine, LMU University Hospital, LMU Munich, Munich, Germany

**Keywords:** Digitalization, Artificial intelligence, Work design, Action regulation theory, Job demands-resources model, Work stress, Motivation

## Abstract

**Background:**

The increasing number of research on algorithmic management (AM) requires measurement tools based on established psychological theories. Filling this gap, the COMpleteness of Algorithmic MAnagement questionnaire (COMAMA) was developed and validated on platform and traditional worker samples in Germany.

**Method:**

The scale was developed in German and its final version also translated into English. Across six samples of workers form various types of occupation (overall *n* = 1005), we tested the content, factorial, discriminant, convergent, differential and criterion validity of the COMAMA scale.

**Results:**

The final 11-item scale assesses the workers perceived takeover of action steps by AM in the action regulation theory-based dimensions of directing, scheduling, monitoring and feedback. These dimensions formed the higher order construct of the completeness of algorithmic management which, as expected, was negatively related to job autonomy and positively related to work pace and irritation.

**Conclusion:**

Overall, the results suggest the COMAMA to be a valid instrument for future research on AM systems and human-centered work design.

**Supplementary Information:**

The online version contains supplementary material available at 10.1186/s40359-025-03680-2.

## Introduction

The present study pursued two main objectives: (1) to develop and validate a novel psychological instrument designed to capture the extent to which Algorithmic Management (AM) assumes control over workers’ action regulation, and (2) to advance the still scarce empirical understanding of how AM affects human-centered work design and employees’ psychological well-being. Algorithmic management (AM) systems are technological systems that have the capability to learn and to make autonomous decisions through algorithmic pattern detection. AM systems are increasingly used to take over typical managerial tasks at work [1–4].

So far, AM systems are mainly implemented to manage work in the so-called new platform economy (e.g., Uber, Amazon MTurk) [5] or in warehouse logistics [6] with rather low skilled jobs. However, AM systems are also beginning to transform traditional organizations and higher qualified jobs, such as engineering [7] and healthcare [8].

Even though AM systems become increasingly important in our work lives, the psychological impact of AM or intelligent technological systems (ITS) in general on work design and mental well-being of employees is not yet well understood [9–14].

This work aims to advance existing research on AM by developing an instrument that can assess possible psychological functions of AM. Such an instrument allows to systematically investigate the relationship between AM, work design and employee well-being. So far, the only instruments that exist today are the *Algorithmic Management Questionnaire* (AMQ) [15] and the *Perceived Algorithmic Control Questionnaire* (PACQ) [16]. Both questionnaires were developed focusing on the organizational tasks AM takes over from human managers. The AMQ distinguishes between *monitoring*, *goal setting*, *scheduling*, *performance rating* and *compensation* [15], while the PACQ distinguishes between *instructive guidance*, *process monitoring* and *feedback evaluation* [16].

As both instruments take the perspective of managerial functions of AM systems within an organization, there is no underlying psychological model used to explain which psychological processes of workers such as action regulation are affected by AM systems. To fill this gap, we take the perspective of action regulation theory to investigate how AM may change workers’ actions required to fulfill their work by choosing dimensions of AM functions substituting or at least affecting the steps of an action sequence of *individual workers* according to action regulation theory. We assume that functions such as reward or discipline (as incorporated in the AMQ [15]) influence employee action more indirectly through broader organizational control mechanisms rather than by directly shaping the sequential steps of individual action regulation. For this reason, these functions fall outside the theoretical scope of COMAMA. By focusing exclusively on the dimensions most directly tied to action regulation, COMAMA provides a parsimonious and at the same time conceptually coherent instrument with clear theoretical interpretability.

## Theoretical foundations

### Action regulation theory

For developing the questionnaire we took the perspective of the action-theoretical concepts *of sequentially and hierarchical complete actions* [17]. This perspective allows to systematically investigate and explain potential psychological functions of AM, and to understand the combined effects of AM functions on the quality of work design and mental well-being across different organizational contexts:

Action Regulation Theory (ART; [17, 18]) captures the cognitive processes of human action regulation and explains their relation to workers’ well-being, personal growth, intrinsic motivation, and good performance. Basically, ART assumes that these desirable individual outcomes are closely intertwined with the extent to which the work environment either promotes or impedes *autonomous actions* of workers by providing scope for decision-making and high levels of personal control.

In ART, actions are seen as sequentially complete when humans not only execute a given activity, but - along a complete action sequence - have also the control to set own goals, plan, and schedule the activity and receive meaningful feedback about the progress of the action [17, 18]. In addition, actions are hierarchically complete, i.e., intellectual stimulating and educational, when they involve not only automated regulation processes, but also knowledge-based and intellectual control processes [17, 18]. Sequentially and hierarchically complete actions are seen as a core feature of humane work design, as they equally increase individual control and promote learning and development at work [17].

As AM potentially can take control over the complete action sequence from the worker, AM is supposed to transfer control about work activities from the individual employee to the organizational system, and with that might have detrimental effects on work design and mental-well-being.

Therefore, we suggest the following working definition of AM: *An algorithmic management system is a form of organizational control which aims to direct the work behavior of employees through rule-based computed (algorithmic) goal setting*,* action-planning*,* scheduling*,* monitoring*,* and feedback*,* without explicit involvement of human managers or other social agents at work. The amount and extent of algorithmic control of these functions indicate the “COMpleteness of Algorithmic MAnagement” (COMAMA)* [19].

Because of the assumed close inherent logical relationship between the single “functions” of AM according to action theory, we believe that they also jointly affect work design and mental well-being and therefore must be studied together and not separately.

### Dimensions of the COMAMA questionnaire

Based on the concept of sequentially complete action [17] and the definition above, the COMAMA questionnaire captures five aspects of algorithmic management *goal setting*, *action planning*, *scheduling*, *monitoring* and *feedback*.

The subscale *goal setting* (GS) assesses the extent to which an AM system specifies work tasks or work objectives for the worker. Goals, in terms of mental representations of future outcomes are one of the fundamental concepts of ART (Hacker, 2003). Goals not only initiate actions but also guide attention throughout the process and serve as criteria for assessing progress. The latitude to set own goals therefore offers the worker autonomy and control about the complete sequence of action [17, 18]. It therefore helps them to avoid overload and negative mental strain [20]. Moreover, it contributes to the perception of more self-determination [21], and higher intrinsic motivation at work [22, 23]. Consequently, externally determined goals through AM should contribute to higher negative mental strain and demotivation [1, 24].

The subscale *action planning* (AP) assesses the extent to which an AM system determines work processes or working methods. Before an action begins, a more or less conscious idea is usually developed as to how the action will be realized, i.e. an action plan is conceived [18]. Action planning is both, an element of job autonomy [25], and also a major regulation requirement [18]. Regulation requirements in ART are primarily seen as motivating and learning-promoting job demands, i.e. challenge demands [26]. On the one hand, coping with these job demands requires individual resources, that will be exhausted at some point and must therefore be regenerated in order to avoid impairment of well-being [27]. On the other hand, however, challenge demands are motivating, as they provide opportunities for learning and their accomplishment meet our basic need for competence [21]. AM systems, however, are often used to provide clear instructions on how a task needs to be done. An Uber driver needs to follow the proposed route [28], and a warehouse logistics worker is being told where to get an item to pack, which box size to use and how long the tape needs to be to seal the package [29]. There are also first examples that AM is also used in higher qualified jobs with more complex work methods like health care [30] and recruiting [31]. As AM relies on the standardization of work, the conceptional aspect of work is shifted from the individual worker to the AM system and thus reduces the amount of individual action planning for the worker [28]. Consequently, externally determined action plans through AM might contribute to decrease negative mental strain, e.g., in terms of physiological and cognitive stress reactions or tension due to high demands, but might at the same time also decrease positive mental strain like activating effects as it contributes to a simplification of work by reducing the cognitive demands and learning opportunities. And thus, leading to more demotivating work [1].

The subscale *scheduling* (SCH) assesses the extent to which an AM system makes specifications about workdays, daily working times, and work breaks. In the broadest sense, scheduling could be seen as part of action planning, that specifically refers to the organization of working time. The possibility to plan your own working time is an important factor for work-life balance and recovery [32]. However, AM systems dynamically adapt action plans and schedules in real time according to current customer and market needs, which makes it harder for the worker to predict their working times [e.g., 5, 33, 34]. This organization and capacity oriented working time flexibility might negatively affect the private life of workers and their opportunities for recovery [32].

The subscale *monitoring* (MO) assesses the extent to which an AM system constantly collects data on work behavior. From the perspective of ART, monitoring one’s own action is the precondition to test whether the action execution leads to the intended goal or deviates from it (e.g. because errors occur) [18]. However, intense electronic monitoring or surveillance can lead to a focus shift and might have negative effects on workers’ intrinsic motivation and well-being [33, 34]: Intense electronic monitoring or surveillance is based on external directives and criteria and may thus lead to decoupling workers from their own action goals, and force them to “work for data” [35]. This means that workers focus on the aspects of a task that are recorded by the monitoring system and less on the things that seem relevant, meaningful and intrinsically motivating to them. Moreover, as electronic monitoring often emphasizes speed and efficiency of action, workers must maintain a constantly high work pace, which increases stressful quantitative job demands [36].

The subscale *feedback* (FB) assesses the extent to which an AM system provides feedback on work behavior and performance. From the perspective of ART, feedback is the result of monitoring. It provides information about the progress of goal attainment and is thus a prerequisite for the successful execution of actions [18]. Therefore, feedback is also a very important source of learning and development at work [17]. AM systems like apps provide workers usually with timely and understandable feedback, e.g., whether a task has been finished or how many tasks have been completed [1]. Such information may strengthen the feeling of workers’ mastery and their experience of competence [24]. However, studies also report that feedback offered by AM systems might not always be helpful: Feedback in AM might be not acceptable to the worker due to the lack of legitimacy of the feedback criteria, and unreliability of the information source, like unjustified reviews from annoyed customers [5, 37]. Moreover, feedback in AM often lacks details and transparency, as workers are frequently unaware of the data used to generate it. Furthermore, the feedback is often aggregated from different data sources [29]. This making it more difficult for workers to identify specific areas for improvement and therefore diminishes the role of feedback as a source of learning. Consequently, feedback provided by AM systems might be understandable but lack credibility and transparency, which might contribute to demotivation of workers and impede learning [38].

Although, as outlined above, each of the individual dimensions of the AM system can have an impact on the quality of work design and the well-being of employees, we also assume holistic effects, that are reflected by the concept *“completeness of AM”.* Particularly, we expect that compared to “traditional” efficiency-driven Taylorist management systems, like the repetitive but predictable work at an assembly line, AM possess entirely new qualities by replacing important social agents at work like human managers, and by making work more opaque and unpredictable in comparison [39].

In addition to an evaluation of the individual subscales, we therefore assume that a joint evaluation of an overall COMAMA score can reveal an independent quality of AM systems that is greater than the sum of its parts. Therefore, the main hypothesis is: *the action regulatory functions of algorithmic management can be mapped with the COMAMA questionnaire.*

### Method

The scale development process consists of six studies with a total of nine different datasets. The datasets are described in detail within their respective studies.

Table 1 provides an overview of all data collected. In the first study the item pool was generated, and the content validity was assessed. The factorial validity was then assessed in two studies with an exploratory factor analysis in study two and a confirmatory factor analysis in study three. The convergent and discriminant validity where both assessed in study four and the differentiation between known groups was tested in study five. Finally, study six assessed the association of the COMAMA scale with work design and negative mental strain. For all studies ethical approval was granted by the responsible committee at the University of Duisburg-Essen prior conducting the research.

**Table 1:** Overview of datasets used

Dataset	Sample size	Study nr.	Data/Study type
Job experts	7	1	Qualitative interviews
I&O psychological experts	10	1	Quantitative survey
Clickworker 1	335	2 & 5	Quantitative survey
Warehouse logistics 1	100	3 & 4	Quantitative survey
Interprofessional sample	273	5	Quantitative survey
Clickworker 2	118	6	Quantitative survey
Warehouse logistics 2	137	6	Quantitative survey
Delivery services	42	6	Quantitative survey

## Scale development

### Study 1: development of the item pool and assessment of the content validity of the COMAMA questionnaire

#### Method

The German item pool was developed using a deductive approach [40]. The baseline for the item wording structure was the “Tätigkeits- und Arbeitsanalyseverfahren (TAA)” [41]. The TAA is a well-established questionnaire to screen for psychological stressors in the work context. The TAA is theoretically based on ART and has been used and validated in different occupational settings [41]. To generate the COMAMA items, the respective items from the TAA were adapted to reflect the AM-specific features. The features of AM at work were derived from qualitative research [1, 29, 42–44]. Care was taken to formulate the items as neutral as possible, to assess AM as independently of subjective bias as possible.

To investigate content validity of the COMAMA questionnaire, we conducted in a first step seven interviews with job experts, i.e., workers who were at least partly managed by algorithms. The aim of the interviews was to examine whether the original pool of 23 items was understandable as well as applicable for assessing the identified functions of algorithmic management.

The participants were recruited in various ways: Digital postings on the website and social media channels of the University of Duisburg-Essen as well as postings on the campus in Essen, Germany. Furthermore, workers were directly addressed at their gathering points, the so-called hubs. A total of seven workers could be recruited to take part in an interview. Three of them were female, while four of them were male. On average the participants were 29.4 years old (SD = 8.3, range 22–43 years). Six participants had advanced level education. Furthermore, all participants had vocational training or a university degree. They had worked on average 4.6 years in their job (SD = 9.0, range 0.1–25 years). The average working time was 19.4 h every week (SD = 12.8, range 7.5–38.5 h every week). The participants predominantly worked in the so-called gig economy. Five worked for food delivery services. Besides that, also one warehouse worker as well as one driver for a large e-commerce company were interviewed. For four of the seven participants, the algorithmically managed job was the main income source. Each interviewee received an online voucher (75€) for participating. A single interview took between 40 and 90 min. The interviews were conducted either at the University of Duisburg-Essen or as a videoconference or telephone conference depending on the participants’ preference.

At the beginning of each interview, the participants were asked to give a brief overview of their job tasks and the implementation of algorithmic management in their jobs. Secondly, participants were presented the identified functions of algorithmic management. They were then instructed to tell whether any of these functions was used in their job If a specific function was used in their job, participants should describe how that function was implemented. After that, participants were asked if there was any function of algorithmic management in their job that was not included in the list of functions presented to them. Afterwards, the participants should outline positive and negative aspects of those algorithmic management functions that were currently used in their jobs.

Ultimately, we conducted cognitive interviews with the job experts [45, 46]. Experts were asked to think-aloud about the items of the questionnaire, i.e., they were actively encouraged to share their thoughts about each question and the appropriateness for measuring algorithmic management. Furthermore, we asked the participants if any function regarding algorithmic management was missing in their opinion.

To evaluate the interviews, we conducted a qualitative content analysis [47] of the answers given by the workers.

In a second step, the adapted version COMAMA questionnaire was sent to experts in the field of industrial and organizational (I&O) psychology. The goal of this part of the validation was to test whether there was an agreement between scientific experts regarding the belonging of the adapted items to the five theoretically derived scales. A total number of ten experts took part in the validation process. The experts had on average 17.2 years of experience in the field of industrial and organizational psychology (*SD* = 8.99, range 4–35 years).

First, the I&O experts were independently asked to assign each item to one of six possible categories. The categories represented the five scales of the COMAMA questionnaire as well as the category “item belongs to no category”, which should be selected if an item would not fit in any of the other categories.

The I&O experts were then asked to rank the items within each scale with regard to the representativeness of individual items for the respective scales. Our goal was to shorten the questionnaire by selecting only the most representative items for each scale. The final 11 items were selected by two criteria. The first criterion was the percental consensus between the raters regarding the assignment of each item to one of the five scales. The second criterion was the median rank for every item in terms of representativeness for the specific scale. To measure the overall interrater reliability of the I-O expert ratings, Fleiss Kappa was used.

#### Results

The interviews with the job experts indicated that all the functions on the scale were considered relevant for their job. Individual items were reformulated to further increase comprehensibility and suitability for the job. Above all, the wording was changed about the degree of binding nature of the instructions by the AM systems (e.g. make specifications vs. determine). Moreover, in item 10 two examples to illustrate work behavior were added. Four additional items were added, primarily reflecting additional aspects of the already predefined functions (e.g., the *number* of work assignments determined by the AM system).

The resulting pool of 27 items were assigned by the I&O experts to the correct scales with 40% to 100% agreement. The goal of the COMAMA development was to have an economic questionnaire with as few items as feasible. Due to the high work pace of the target group of this questionnaire we consider it important to reduce the time required to answer it to a minimum. The more time a worker must spend on a survey, the more likely it is that the survey is not completed. Additionally, there is growing evidence that short psychological measures with even just one indicator, if well-constructed, can be just as valid and reliable as scales with multiple indicators [48]. Therefore, we selected only those two items for each of the five scales with the highest agreement respectively. An exception was the scheduling scale where we chose to keep all three items because each represented a unique aspect of scheduling (the results of the validation presented in the following studies indicate that the psychometric properties of the scale were also satisfactory with two items each). If two items had the same percental consensus, the item with the higher median rank in terms of representativeness for the scale was chosen. The lowest agreement of the finally selected 11 items had the items 1 and 2 (respectively 80% agreement) that were originally intended to represent goal setting. Fleiss Kappa score for the correct assignment of the 11 items was 0.754, which can be interpreted as substantial agreement [49]. All items are to be answered on a five-point Likert scale containing the responses: (1) not at all, (2) rather no, (3) partly, (4) rather yes, (5) yes, exactly. The final version of the COMAMA scale can be found in Appendix I.

### Study 2: assessment of factorial validity (Exploratory Factor Analyses, EFA)

#### Method

To analyze the factorial structure of the items, we collected data via the online platform clickworker.de, a digital labor platform based in Germany with a large user base. These clickworkers work as freelancers and perform microtasks like training of artificial intelligence systems. To improve the data quality bogus items (i.e., instructed response items) were used. After removing all participants who failed the attention checks with bogus items, a total of 335 participants was used for the EFA. 41.5% of the participants were female, the mean age was 38.93 years (*SD* = 11.75). 49.3% stated to have only the one job as a clickworker. Participants were asked to what degree they agreed with each item on a five-point Likert scale (1 = not at all, 5 = yes, exactly). It was ensured that participants could not complete the survey more than once. Every participant received 4€ after completing the survey. Two common criteria for determining the number of factors were used [50]: The number of eigenvalues >1 and the number of eigenvalues that cumulatively account for 80% of total variance. An oblique rotation was used as the psychological functions of AM represent different stages of the action sequence according to ART and thus are not independent. The EFA was performed using the psych package in R 4.4.

#### Results

The Kaiser-Meyer-Olkin test (KMO) indicated that the collected data was suitable for conducting the EFA (*KMO* = 0.85). The Bartlett’s test of sphericity was significant (*X*^*2*^ = 2140.786, *Df* = 55, *p* <.000). The number of eigenvalues > 1 was three, suggesting a three-factor structure, four eigenvalues are needed to explain a cumulative percentage of variance of 80%, suggesting a four-factor structure, whereas a 5-factor structure represents our above-described assumption based on ART. We therefore decided to perform the EFA for five (to test the theoretical model), four (following the 80% variance rule) and three factors (following the eigenvalue > 1 rule, to compare the results.

The 5-factor EFA showed cumulative variance accounting for 66.7% of the total variance. The resulting factor loadings correspond with the theoretical factor model except item AP1, which shows main loadings on the same factor as the GS-items. The internal consistency was *α* = 0.87 for the total scale, showing good internal consistency in most factors: *goal setting* (GS): *α* = 0.86; *action planning* (AP): *α* = 0.82; *scheduling* (SCH): *α* = 0.88; *monitoring* (MO): *α* = 0.66; *feedback* (FB): *α* = 0.79). The intercorrelations of the COMAMA dimensions are presented in Table 2.

**Table 2:** Means, standard deviations, and correlations of the 5-factor model

Variable	M	SD	1	2	3	4	5
1. Goal setting	3.36	1.15					
2. Action planning	3.33	1.12	0.78**				
3. Scheduling	2.07	1.09	0.29**	0.32**			
4. Monitoring	1.98	0.98	0.26**	0.31**	0.53**		
5. Feedback	2.37	1.05	0.32**	0.37**	0.42**	0.61**	
6. COMAMA	2.57	0.79	0.71**	0.74**	0.76**	0.72**	0.72**

M and SD are used to represent mean and standard deviation, respectively

* indicates *p* <.05. ** indicates *p* <.01

The 4-factor EFA explained a cumulative variance of 65.9%. The items for goal setting and action planning showed loadings above 0.7 on the same factor. The item MO1 did not show a loading above 0.35 on any of the four factors. As the items MO1 and MO2 are intended to measure two different aspects of monitoring (work performance and social interaction with colleagues), the item MO1 was kept in the questionnaire. The internal consistency was *α* = 0.87 for the total scale, showing good internal consistency in most factors: *goal setting & action planning* (GS&AP): *α* = 0.90; *scheduling* (SCH): *α* = 0.88; *monitoring* (MO): *α* = 0.66; *feedback* (FB): *α* = 0.79). The intercorrelations of the COMAMA dimensions are presented in Table 3.

**Table 3:** Means, standard deviations, and correlations of the 4-factor model

Variable	M	SD	1	2	3	4
1. Goal setting & action planning	3.35	1.07				
2. Scheduling	2.07	1.09	0.32**			
3. Monitoring	1.98	0.98	0.30**	0.53**		
4. Feedback	2.37	1.05	0.36**	0.42**	0.61**	
5. COMAMA	2.57	0.79	0.77**	0.76**	0.72**	0.72**

M and SD are used to represent mean and standard deviation, respectively

* indicates *p* <.05. ** indicates *p* <.01

The 3-factor EFA explained a cumulative variance of 62.5%. In addition to the combination of goal setting and action planning, monitoring and feedback items showed loadings above 0.5 on the same factor. The internal consistency was *α* = 0.87 for the total scale, showing good internal consistency in most factors: *goal setting & action planning* (GS&AP): *α* = 0.90; *scheduling* (SCH): *α* = 0.88; *monitoring & feedback* (MO&FB): *α* = 0.80). The intercorrelations of the COMAMA dimensions are presented in Table 4.

**Table 4:** Means, standard deviations, and correlations of the 3-factor model

Variable	M	SD	1	2	3
1. Goal setting & action planning	3.35	1.07			
2. Scheduling	2.07	1.09	0.32**		
3. Monitoring & Feedback	2.17	0.91	0.37**	0.53**	
4. COMAMA	2.57	0.79	0.77**	0.76**	0.80**

M and SD are used to represent mean and standard deviation, respectively

* indicates *p* <.05. ** indicates *p* <.01

The results of the exploratory factor analysis are presented in Table 5.

**Table 5:** EFA factor loadings for 5, 4 and 3 factors

	5-factor model					4-factor model				3-factor model		
	GS	AP	SCH	MO	FB	GS&AP	SCH	MO	FB	GS&AP	SCH	MO&FB
Eigenvalue	4.86	0.49	2.16	0.60	1.21	4.86	2.16	0.60	1.21	4.86	2.16	1.21
Variance	0.182	0.096	. 178	0.101	0.110	0.253	0.182	0.097	0.127	0.255	0.192	0.178
GS1	**0.906**					**0.850**				**0.831**		
GS2	**0.872**					**0.885**				**0.861**		
AP1	**0.641**	0.199				**0.833**				**0.840**		
AP2		**0.983**				**0.737**			0.121	**0.756**		0.102
SCH1			**0.868**				**0.871**				**0.852**	
SCH2			**0.962**				**0.970**				**0.950**	
SCH3			**0.523**	0.331			**0.547**	0.266			**0.616**	0.293
MO1		0.200		**0.391**	0.217	0.195		0.282	0.319	0.163		.**535**
MO2				**0.866**				**0.940**		− 0.161	0.298	**0.599**
FB1				0.304	**0.573**			0.144	**0.734**			.**849**
FB2					**0.913**				**0.850**	0.172		.**706**

*N* = 335. Factor loadings above 0.35 are in bold

### Study 3: assessment of factorial validity (Confirmatory Factor Analyses, CFA) model specification and internal consistency

#### Method

We conducted a confirmatory factor analysis CFA to assess the fit of the hypothesized factorial structure to the data. For this purpose, we collected data from workers in the logistics sector using the survey panel provider Bilendi. The logistics sector is one of the first traditional industries to adopt the usage of AM systems [51]. Bilendi used their “niche-sampling” technique to reach the required target group via social media. Screening questions ensured only workers in warehouse logistics working more than 20 h per week participated. To improve the data quality, bogus items were used. The sample used for the CFA was composed of 100 workers of which 72% worked 40 h and more per week. 42% worked as logistician, 13% as warehouse helper, 8% as driver, 8% as forklift driver.

Based on the results of the EFA, we adapted our assumed model, consisting of five factors and one higher order factor, by shifting the item AP1 to the *goal setting* factor (model 1). The second model proposes that the factors *goal setting* and *action planning* cannot be differentiated but measure just one factor called *directing*. The AMQ [15] is an example of a questionnaire where *goal setting* and *action planning* are combined into one factor; Fernández-Macías, Urzì Brancati [52] as well propose one combined factor *direction.* This resulted in four factors and one higher order factor (model 2). The third model proposes three-factors model according to the EFA 3 factor solution and one higher order factor (model 3). It is based on model 2 with additionally combining the factors *monitoring* and *feedback* into *evaluating*. The fourth model (model 4) assumes one general AM factor without subfactors. The CFA was performed using the lavaan package in R 4.4. The goodness of fit was assessed using the rule of thumb indicators [53]: (a) comparative fit index (CFI; target: >0.90), (b) Tucker–Lewis index (TLI; target >0.90), (c) root mean square error of approximation (RMSEA < 0.08), and (d) standardized root mean square residual (SRMR; target < 0.08). The Mardia’s test indicated a significant deviation from a multivariate normal distribution. Thus, we used the Satorra Bentler correction [54].

#### Results

**Fig. 1:**
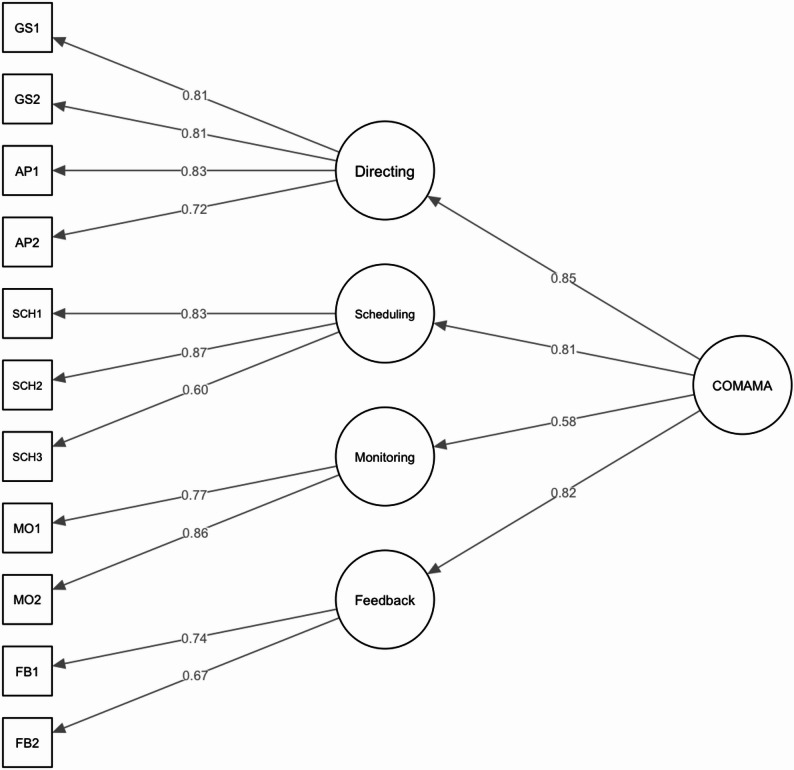
Final measurement model

Model 1 and model 2 both showed good fit to the data and were superior to all other models. Both models would be acceptable from a theoretical point of view. And although it has been shown that one-item-scales – as contained in model 1 – do not need to be considered inferior to multi-item-scales [48] we chose model 2 with four factors for a more robust scale. This led us to adapt our factor naming combining the factors *goal setting* and *action planning* into *directing* (DI). The internal consistency was *α* = 0.87 for the total scale, showing good internal consistency in most factors: *directing* (DI): *α* = 0.87; *scheduling* (SCH): *α* = 0.81; *monitoring* (MO): *α* = 0.78; *feedback* (FB): *α* = 0.66). The intercorrelations of the COMAMA dimensions are presented in Table 6. The final measurement model is shown in Fig. 1, the model comparison results are shown in Table 7.

**Table 6:** Means, standard deviations, and correlations

Variable	M	SD	1	2	3	4
1. Directing	3.35	1.07				
2. Scheduling	2.07	1.09	0.32**			
3. Monitoring	1.98	0.98	0.30**	0.53**		
4. Feedback	2.37	1.05	0.36**	0.42**	0.61**	
5. COMAMA	2.57	0.79	0.77**	0.76**	0.72**	0.72**

M and SD are used to represent mean and standard deviation, respectively

* indicates *p* < .05. ** indicates *p* < .01

**Table 7:** CFA model comparison

Model	df	X^2^	CFI	TLI	RMSEA [90% CI]	SRMR	AIC	BIC
Model 1	31	43.847	0.959	0.941	0.076 [0.000 − 0.124]	0.055	2617.477	2680.001
Model 2	40	43.789	0.987	0.982	0.039 [0.000 − 0.097]	0.056	2841.035	2908.769
Model 3	41	58.598*	0.938	0.917	0.084 [0.022 − 0.130]	0.078	2866.760	2931.890
Model 4	44	104.609**	0.791	0.739	0.149[0.112 − 0.186]	0.096	2932.681	2989.994

*N* = 100. Reported statistics are Satorra Bentler corrected

*AIC *Akaike information criterion*, BIC *Bayesian information criterion*, CFI *confirmatory fit index*, df *degrees of freedom*, RMSEA *root mean square error of approximation*, SRMR *standardized root mean square residual*, TLI *Tucker-Lewis index

* indicates *p* <.05. ** indicates *p* <.001

### Study 4: convergent and discriminant validity

#### Method

To check the construct validity of the COMAMA scale, we looked at the convergent and discriminant validity and at the differentiation between known groups.

Convergent validity is the extent to which a construct measured in different ways yields similar results. Specifically, it is the “degree to which scores on a studied instrument are related to measures of other constructs that can be expected on theoretical grounds to be close to the one tapped into by this instrument” [55]. For this we used the Algorithmic Management Questionnaire (AMQ) [15]. We translated the items into German following the method proposed by Harkness [56]. The scales and items were independently translated by two researchers (German native speakers who are fluent in English) that were acquainted with the theoretical background of the AMQ. Additionally, a third translation was created by using the software deepl. In a second step, all three translations were compared and differences that had occurred in the translations were discussed. As a result of the comparison and discussion process, the final German version of the AMQ was used in the “warehouse logistics 1” sample (*n* = 100). We expected to find moderate to high correlations between the AMQ and the COMAMA scale. Discriminant validity is the extent to which a measure is novel and not simply a reflection of some other construct [55]. We tested discriminant validity via correlations between the COMAMA scale with the pragmatic quality of user experience scale (UEQS) [57]. We expected low correlations as the user experience of an information technology system is a different feature as the actions steps taken over by that same system.

#### Results

As shown in Table 8, the COMAMA scale correlated highly (0.71) with the AMQ. Although the COMAMA and the AMQ are different in their theoretical foundation and therefore measure different constructs, their ability to measure the exposure to AM systems correlate well (shared variance about 50%). These results indicate that the COMAMA scale presented good convergent validity. The internal consistency was *α* = 0.88 for the COMAMA scale, *α* = 0.95 for the AMQ scale and *α* = 0.96 for the UEQS scale.

Table 8 also shows the correlations for the COMAMA scale with the UEQS being low (0.23) supporting the assumption of discriminant validity.

**Table 8:** Descriptive statistics and correlations for convergent and discriminant validity

Variable	M	SD	1	2	3	4	5	6
1. User experience	4.24	0.85						
2. AMQ	1.70	0.99	0.15					
3. Directing	2.11	1.16	0.20*	0.60**				
4. Scheduling	1.42	0.73	0.20*	0.56**	0.59**			
5. Monitoring	1.71	1.07	0.15	0.46**	0.39**	0.32**		
6. Feedback	1.51	0.79	0.12	0.58**	0.54**	0.46**	0.40**	
7. COMAMA	1.74	0.76	0.23*	0.71**	0.91**	0.76**	0.63**	0.71**

M and SD are used to represent mean and standard deviation, respectively

* indicates *p* <.05. ** indicates *p* <.01

### Study 5: differentiation between known groups

#### Method

Differentiation or comparison between known groups examines the distribution of a newly developed scale score over known binary items [55] For this purpose, we collected an interprofessional sample of employees with a wide spectrum of occupations. The participants were recruited by direct approach and via social media and under all participants two vouchers of 50€ each were raffled. After deleting participants with failed attention checks and incomplete data the resulting sample contained 273 participants (67% female, 63% full-time employed with an average age of 38.98 years). This sample was compared to the “clickworker 1” sample (*n* = 335). We expected the “clickworker 1” sample to show higher scores in all COMAMA factors and the overall scales than the “interprofessional” sample (*n* = 273).

#### Results

The results shown in Table 9 confirm the differences in the mean scores between these two samples. As expected, all AM functions as well as the degree of total AM were significantly higher in the clickworker sample. The subscales Scheduling and Monitoring show a moderate effect size while Directing, Feedback and the total scale had a large effect size [58]. The internal consistency for the clickworker sample was *α* = 0.87 for the total scale, showing good internal consistency in most factors: *directing* (DI): *α* = 0.90; *scheduling* (SCH): *α* = 0.88; *monitoring* (MO): *α* = 0.66; *feedback* (FB): *α* = 0.79). The internal consistency for the interprofessional sample was *α* = 0.85 for the total scale, showing good internal consistency in most factors: *directing* (DI): *α* = 0.84; *scheduling* (SCH): *α* = 0.83; *monitoring* (MO): *α* = 0.71; *feedback* (FB): *α* = 0.80).

**Table 9:** Overview over mean differences in the scales between “clickworker sample” and “interprofessional sample”

	Clickworker sample (*n* = 335)		Interprofessional sample (*n* = 273)				
	*M*	*SD*	*M*	*SD*	*t*(606)	*p*	*d*
Directing	3.35	1.07	2.35	1.05	11.537	0.000	0.941
Scheduling	2.07	1.09	1.53	0.90	6.644	0.000	0.537
Monitoring	1.98	0.98	1.47	0.79	7.081	0.000	0.571
Feedback	2.37	1.05	1.36	0.70	14.119	0.000	1.129
COMAMA	2.57	0.79	1.79	0.67	13.240	0.000	1.071

### Study 6: criterion validity - expected association of AM with work design and negative mental strain

According to our introductory considerations, we examined the association of the COMAMA scale with health-relevant work design characteristics [1, 19] and negative mental strain [59].

#### Work pace

AM increases work pace by continually optimizing work efficacy [60, 61]. By setting performance targets based on past data, it fosters ever-rising productivity standards [62]. In warehouses, scanners display daily targets, enforced by supervisors [29]. AM monitors task performance extensively, using data to nudge worker behavior. On some platforms, it adjusts pay rates in real time, pushing workers to chase lucrative but irregular hours, often at the expense of free time [43, 63, 64]. Additionally, AM’s feedback changes constantly as algorithms incorporate real time data [65]. We thus formulated the following hypothesis:

- *Hypothesis 1: More complete AM is associated with higher work pace.*

#### Job autonomy

More complete AM systems are linked to reduced job resources [1]. Across different work settings, AM predefines task goals, limiting worker influence. Unlike traditional management, where supervision usually allows for individual task planning, AM’s strict task assignment and monitoring hinder autonomy [66, 67]. Algorithms minimize human intervention, making task negotiation difficult. In platform work, frequent task rejection is often sanctioned [5, 60, 68–70]. Additionally, AM’s reliance on quantification encourages “working for data,” further reducing autonomy. Thus, more complete AM restricts job autonomy, a key resource for action regulation in ART [18] leading to the following hypothesis:

- *Hypothesis 2: More complete AM is associated with lower job autonomy.*

#### Negative mental strain

AM has been reported to affect the mental well-being of workers in a negative way [4, 59]. On the one hand AM is expected to reduce job resources and increase job demands [1, 19]. In line with the Job Demands-Resources Model (JD-R model) this is associated with reduced mental well-being and increased negative strain experience [24]. On the other hand it has been found that the facilitation of control through AM systems also corresponds directly with threat techno-stressors [71]. We therefore propose the following hypothesis:

- *Hypothesis 3: More complete AM is associated with higher negative mental strain.*

#### Method

The data we use to assess the criterion validity was gathered in three separate surveys capturing three different groups of algorithmically managed workers. In the first sample, we again used the platform clickworker.com. After removing the participants not meeting the quality checks 118 participants remained in the “clickworker 2” sample. 64.4% were male and 67.8% were younger than 40 years. The “warehouse logistics 2” sample addressed workers in warehouse logistics using the company Bilendi to sample the data. 137 participants passed the data quality assurance with 75.2% being male and 24.8% being younger than 40 years old. The “delivery services” sample covered delivery services and was gathered personally. 42 participants were kept after quality control with 92.9% being male and 61.9% being younger than 40 years. Thus, the combined dataset contained 297 participants. All three samples were based on surveys that do not overlap with previously reported samples.

Work pace was measured by three items from the *Copenhagen Psychosocial Questionnaire* [72]. A representative item is “*Do you have to work very fast?*” Cronbach’s α was 0.85 in our study.

Job autonomy was measured by three items from the decision-making autonomy sub scale from the *Work Design Questionnaire* [25]. A representative item is “*The job allows me to make a lot of decisions on my own.*” Cronbach’s α was 0.88 in our study.

Negative strain experience was measured with the eight items of the *Irritation Scale for the Assessment of Work-Related Strain* [73]. A representative item is “*Even at home I often think of my problems at work.*” Cronbach’s α was 0.92 in our study.

We controlled for participants’ gender, age (2 = 18–29 years, 3 = 30–39 years, 4 = 40–49 years, 5 = 50–59 years, 6 = 60–69 years, 7 = 70 years and above) and job type (clickworker, warehouse logistics and delivery services).

A structural equation model was calculated to evaluate the associations between COMAMA and the variables described above using the lavaan package in R 4.4.1.

#### Results

We first performed a CFA to check the fit of the COMAMA measurement model which showed a good fit (*X*^2^ = 94.427, df = 40, CFI = 0.974, TLI = 0.964, RMSEA = 0.078, SRMR = 0.051). The internal consistency was *α* = 0.90 for the COMAMA scale, *α* = 0.85 for the work pace scale, *α* = 0.88 for the job autonomy scale and *α* = 0.92 for the irritation scale.

The correlations and descriptive statistics are shown in Table 10. The direction of COMAMA correlations were as expected, showing positive correlations with work pace and irritation and a negative correlation with job autonomy. The subscale monitoring had the highest correlation with work pace and irritation, job autonomy showed the highest correlations with directing and monitoring. The indications observed in the correlations were further supported by a structural equation model (SEM).

**Fig. 2:**
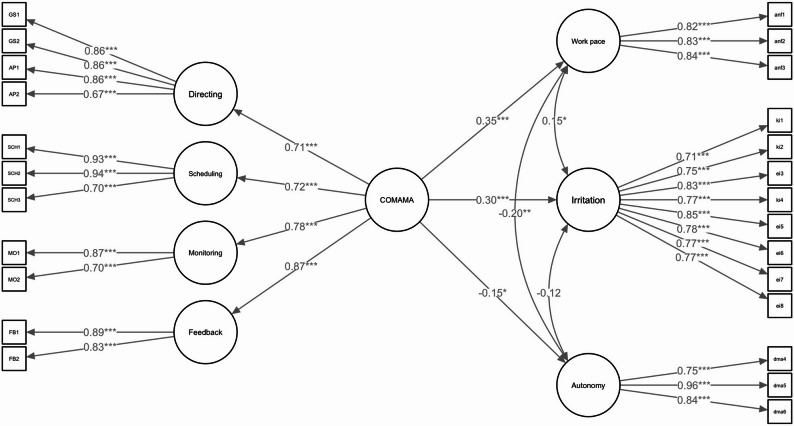
Structural equation model. Note. The effects of control variables *gender*, *age* and *sample* are not shown for reasons of clarity

The complete SEM (Fig. 2) showed an acceptable fit (*X*^2^ = 934.610, df = 354, CFI = 0.871, TLI = 0.855, RMSEA = 0.080, SRMR = 0.100). The results of the SEM are shown in Table S1 in Appendix III. For clarity’s sake, control variables are omitted from the figure.

COMAMA is positively associated with work pace (*ß* = 0.351, *p* <.001), negatively associated with job autonomy (*ß* = − 0.148, *p* <.05) and - independently from the two work design measures - positively associated with irritation (*ß* = 0.301, *p* <.001). These results are in full support of the formulated hypotheses.

**Table 10:** Means, standard deviations and correlations

Variable	M	SD	1	2	3	4	5	6	7	8	9	10	11	12
1. Gender (= female)	0.26	0.44												
2. Age	3.69	1.25	0.02											
3. Clickworker (= yes)	0.40	0.49	0.16**	− 0.28**										
4. Logistics (= yes)	0.46	0.50	− 0.04	0.38**	− 0.75**									
5. Delivery (= yes)	0.14	0.35	− 0.17**	− 0.14*	− 0.33**	− 0.38**								
6. Work pace	3.16	0.94	0.06	0.04	− 0.07	0.08	− 0.02							
7. Autonomy	3.28	1.07	− 0.04	0.00	− 0.05	0.12*	− 0.10	− 0.20**						
8. Irritation	21.13	9.63	− 0.02	− 0.15*	0.19**	− 0.07	− 0.17**	0.18**	− 0.11					
9. Directing	2.95	1.22	− 0.02	− 0.31**	0.40**	− 0.47**	0.12*	0.08	− 0.14*	0.11				
10. Scheduling	2.01	1.14	0.02	− 0.33**	0.28**	− 0.38**	0.16**	0.13*	− 0.05	0.23**	0.48**			
11. Monitoring	2.20	1.15	− 0.08	− 0.25**	0.15**	− 0.27**	0.17**	0.21**	− 0.14*	0.33**	0.40**	0.53**		
12. Feedback	2.21	1.22	− 0.01	− 0.32**	0.42**	− 0.52**	0.16**	0.11	− 0.13*	0.18**	0.60**	0.57**	0.54**	
13. COMAMA	2.42	0.95	− 0.02	− 0.39**	0.40**	− 0.53**	0.18**	0.15**	− 0.14*	0.24**	0.85**	0.80**	0.71**	0.82**

Control variables 1 to 5 are dummy coded (1 = yes)

* indicates *p* <.05. ** indicates *p* <.01

## Discussion

The aim of the research presented in this paper was twofold: (1) to develop and validate a new psychological measure to specifically assess the amount of control over worker´s action regulation taken over by Algorithmic Management (AM) and (2) to contribute to the currently limited empirical evidence on the impact of AM on human-centered work design and employees’ psychological well-being. Therefore, we created the *COMpleteness of Algorithmic MAnagement* questionnaire (COMAMA) drawing on action regulation theory [17]. The results of six studies using qualitative (i.e., expert interviews) and quantitative (i.e., online surveys) methods led us to conclude that the COMAMA scale presents good factorial, discriminant and convergent validity. We identified a four-factor structure as the most feasible solution balancing theoretical foundation and capability to differentiate subfactors. We could show that COMAMA is not an instrument to assess the user experience of technology but rather a tool to measure the exposure to AM. Furthermore, results suggest that COMAMA is able to show associations to work design outcomes as proposed by the current state of research [1, 3, 19, 24].

The items of the scale distinctively capture four action relevant functions of AM: directing, scheduling, monitoring, and feedback, with these functions reflecting a higher order construct of exposure to AM. Moreover, we found indications that the completeness of algorithmic management is associated with an increase in job demands (i.e., work pace) and a decrease in job resources (i.e., job autonomy) as well as a decrease in mental well-being (i.e., higher levels of irritation). In line with prior research on AM [1, 24], these findings also align with the Job Demands-Control Model [20], which posits that high job demands combined with low job control can lead to increased negative mental strain and adverse mental health outcomes. As such, our results suggest that more comprehensive algorithmic management systems may contribute to stressful work environments by intensifying demands while simultaneously undermining workers’ autonomy, thereby exacerbating risks to mental health [74]. As most of the existing research is of qualitative nature, our studies complement the current body of knowledge with quantitative data.

To the best of our knowledge, the COMAMA questionnaire is the first instrument measuring exposure to AM based on an established psychological theory. This strictly theory-driven approach enabled us to develop an instrument that is independent of the specific work environment or job type. During the development of COMAMA, data samples from jobs in the platform economy as well as traditional workplaces (e.g., warehouse logistics) were used to ensure its applicability across different job types. Although there are certain commonalities with existing measures like the AMQ [15], the COMAMA is based on the perspective on workers’ actions and not exclusively of the features of an AM system.

This perspective assumes that the psychological effects of AM can be explained by the channeling of human action regulation through these systems [19]. It offers a fruitful foundation for further work and organizational psychology research in this field, as it allows for the development of hypotheses that can be empirically tested using the COMAMA scale. We therefore believe that COMAMA will help researchers to further investigate and understand the impairment of workers’ mental well-being by AM systems in their current way of implementation. In addition, the deeper understanding of the psychological effects of AM on workers will hopefully enable AM developers to consider more human-centered design approaches in the future to utilize AM advantages without impairing employee’s health and wellbeing. By integrating ART and AM research we hope to contribute to an increased awareness of ART within the AM and artificial intelligence academia. Furthermore, we hope that COMAMA will be used to apply ART research to new job types like the ones within the platform economy.

### Limitations and future research

More research is needed to test the applicability and validity of the instrument in different contexts. Scholars emphasize that the psychosocial effects of technological systems like AM are not uniform but depend on external factors such as the organizational context [2, 10]. For example, platform organizations and “regular” organizations differ regarding the employment status of workers, which may also significantly impact workers’ rights. In regular organizational settings, legal protections and co-determination rights may therefore mitigate some of AM’s adverse effects. Platform-based organizations may offer greater flexibility and autonomy regarding work schedules. In both traditional and platform organizations, variations may exist in terms of total working hours and the degree of financial dependence on the work (e.g., primary source of income versus supplementary earnings). Thus, more research is needed to fully understand potential contextual differences of psychosocial AM effects. Furthermore, we only used cross-sectional data to validate the COMAMA, that do not allow to draw causal inferences. Moreover, observed associations may be inflated due to common method bias [75] which might play a relevant role specifically in the test for criterion validity where independent and dependent variables are assessed at the same time via self-report. However, we believe that characteristics of AM are likely to be only minimally susceptible to distortions caused by individual response tendencies, as they capture factual conditions (e.g. “Information technologies (e.g., software, apps) make specifications, which tasks or assignments I have to complete.”), and “only” medium-sized associations (Table 10; Fig. 2) support this notion. Moreover, for this same reason, potential reverse causal relationships (e.g., that negative mental strain “causes” AM) appear implausible. Nonetheless, future longitudinal studies are both meaningful and desirable—to examine how negative (and positive) mental strain under algorithmic management develops over time.

Lastly, we developed and validated the COMAMA questionnaire with German workers only. Further research needs to investigate the applicability and validity of other languages in other countries.

### Conclusion

With the COMAMA questionnaire we provide a validated instrument, based on established psychological theory, to investigate the impact on AM on human-centered work design and workers mental well-being. This questionnaire enables researchers to gain a more differentiated psychological understanding of the effects of AM in different work environments, and to support the human-centered implementation of AM in the future.

## Supplementary Information

Supplementary Material 1.

Supplementary Material 2.

Supplementary Material 3.

## Data Availability

The data for this article are available on request to the corresponding author.

## Abbreviations

AIC: Akaike information criterion
AM: Algorithmic Management
AMQ: Algorithmic Management Questionnaire
AP: Action planning
ART: Action Regulation Theory
BIC: Bayesian information criterion
CFA: Confirmatory factor analyses
CFI: Comparative fit index
COMAMA: Completeness of algorithmic management
DI: Directing
EFA: Exploratory factor analyses
FB: Feedback
GS: Goal setting
I&O: Industrial and organizational
ITS: Intelligent technological systems
JD-R model: Job Demands-Resources Model
KMO: Kaiser-Meyer-Olkin test
MO: Monitoring
PACQ: Perceived Algorithmic Control Questionnaire
RMSEA: Root mean square error of approximation
SCH: Scheduling
SEM: Structural equation model
SRMR: Standardized root mean square residual
TAA: Tätigkeits- und Arbeitsanalyseverfahren
TLI: Tucker–Lewis index
UEQS: User experience scale
