# Supplementary material for: Algorithmic management: psychological measurement and associations with work design and mental strain
Source: BMC Psychol. 2025 Dec 1;13:1327. doi: 10.1186/s40359-025-03680-2 (PMC12670828; doi:10.1186/s40359-025-03680-2)
Supplement: Supplementary file 1 — Supplementary Material 1. [file 40359_2025_3680_MOESM1_ESM.docx]

# Appendix I

## German version of the COMAMA questionnaire

1. Informationstechnologien (z.B. Software, Apps) machen Vorgaben, welche Aufgaben oder Arbeitsaufträge ich zu erledigen habe. (DI)
2. Informationstechnologien (z.B. Software, Apps) geben mir Arbeitsziele vor. (DI)
3. Mein Arbeitsablauf wird durch Informationstechnologien (z.B. Software, Apps) festgelegt. (DI)
4. Informationstechnologien (z.B. Software, Apps) legen fest, welche Arbeitsmethoden ich einsetze. (DI)
5. Informationstechnologien (z.B. Software, Apps) machen Vorgaben an welchen Tagen ich arbeite. (SCH)
6. Informationstechnologien (z.B. Software, Apps) machen Vorgaben, zu welchen Tageszeiten ich arbeite. (SCH)
7. Informationstechnologien (z.B. Software, Apps) machen Vorgaben, wann ich Pause mache. (SCH)
8. Bei meiner Arbeit werden fortlaufend Daten (z.B. Bewegungsdaten, Internetaktivitäten, Videoaufzeichnungen) darüber aufgezeichnet, wie ich meine Arbeit erledige. (MO)
9. Bei meiner Arbeit werden fortlaufend Daten (z.B. Bewegungsdaten, Internetaktivitäten, Videoaufzeichnungen) darüber aufgezeichnet, wie ich mit Kolleg:innen zusammenarbeite. (MO)
10. Informationstechnologien (z.B. Software, Apps) geben mir Rückmeldung zu meinem Arbeitsverhalten (z.B. zu meiner Schnelligkeit, Freundlichkeit). (FB)
11. Informationstechnologien (z.B. Software, Apps) geben mir Rückmeldung, wie gut ich meine Arbeit erledigt habe. (FB)

*Anmerkung.* DI = Skala „Zielsetzung und Handlungsplanung“, SCH = Skala „Arbeitszeitplanung“, MO = Skala „Monitoring“, FB = Skala „Feedback. Fünfstufige Skalierung mit den Antwortstufen: (1) nein, gar nicht, (2) eher nein,
(3) teils, teils, (4) eher ja, (5) ja, genau.
