# Supplementary material for: Algorithmic management: psychological measurement and associations with work design and mental strain
Source: BMC Psychol. 2025 Dec 1;13:1327. doi: 10.1186/s40359-025-03680-2 (PMC12670828; doi:10.1186/s40359-025-03680-2)
Supplement: Supplementary file 2 — Supplementary Material 2. [file 40359_2025_3680_MOESM2_ESM.docx]

# Appendix II

## Translation process of the English version of the COMAMA questionnaire

For translating the questionnaire we followed the method proposed by Harkness [1]. The first step of the translation process was to produce two separate translations of the COMAMA questionnaire. Two researchers (German native speakers who are fluent in English) that were familiar with the theoretical background of the questionnaire translated the items and scales independently. The validated English items of the “Tätigkeits- und Arbeitsanalyseverfahren (TAA)” were used to as starting point for the translation [40].

The second step was to compare both translations and discuss differences that occurred in the translations. As a result of the comparison and discussion process, a preliminary version of the English translation was developed.

In a third step, this preliminary version was sent to an English language expert (teacher at a German grammar school). The expert made final corrections and was able to answer questions regarding the translation that had remained unanswered after the comparison discussion process. The result is the final version of the English COMAMA questionnaire.

## English version of the COMAMA questionnaire

1. Information technologies (e.g., software, apps) make specifications, which tasks or assignments I have to complete. (DI)
2. Information technologies (e.g., software, apps) set my work goals. (DI)
3. My work process is determined by information technologies
   (e.g., software, apps). (DI)
4. Information technologies (e.g., software, apps) determine which working methods I use. (DI)
5. Information technologies (e.g., software, apps) make specifications about the days on which I work. (SCH)
6. Information technologies (e.g., software, apps) make specifications about the times of the day which I work at. (SCH)
7. Information technologies (e.g., software, apps) make specifications about when I take breaks. (SCH)
8. At my work, data (e.g., movement data, internet activities, video recordings) is constantly assessed on how I carry out my work. (MO)
9. At my work, data (e.g., movement data, internet activities, video recordings) is continuously assessed about how I collaborate with colleagues. (MO)
10. Information technologies (e.g., software, apps) give me feedback on my work behavior (e.g., my speed, friendliness). (FB)
11. Information technologies (e.g., software, apps) give me feedback on how well I did my work. (FB)

*Note.* DI = scale “directing”, SCH = scale “scheduling”, MO = scale “monitoring”, FB = scale “feedback. Five-point scale containing the responses: (1) not at all, (2) rather no, (3) partly, (4) rather yes, (5) yes, exactly.
