# Supplementary material for: Algorithmic management: psychological measurement and associations with work design and mental strain
Source: BMC Psychol. 2025 Dec 1;13:1327. doi: 10.1186/s40359-025-03680-2 (PMC12670828; doi:10.1186/s40359-025-03680-2)
Supplement: Supplementary file 3 — Supplementary Material 3. [file 40359_2025_3680_MOESM3_ESM.docx]

**Appendix III**

**Table S1**

Criterion validity results

|  | Work pace |  | Autonomy |  | Irritation |
| --- | --- | --- | --- | --- | --- |
| Variable | Est. |  | Est. |  | Est. |
| Gender | .086 |  | -.070 |  | -.037 |
| Age | .077 |  | -.099 |  | -.087 |
| Clickworker | -.232*** |  | -.063 |  | -.011 |
| Delivery | -.136 |  | -.115 |  | -.252*** |
| COMAMA | .351*** |  | -.148* |  | .301*** |
| *R*^2^ | .199 |  | .041 |  | .152 |

*Note.*. * indicates *p* < .05. ** indicates *p* < .01. *** indicates *p* < .001. The reference group gender is male. The reference group of the three samples is the logistics sample
